# Yeast Synthetic Biology for the Production of *Lycium barbarum* Polysaccharides

**DOI:** 10.3390/molecules26061641

**Published:** 2021-03-15

**Authors:** Jinjin Peng, Luan Wang, Mengge Wang, Rui Du, Shangshang Qin, Cheng-Yun Jin, Yongjun Wei

**Affiliations:** 1 Key Laboratory of Advanced Drug Preparation Technologies, School of Pharmaceutical Sciences, Zhengzhou University, Ministry of Education, Zhengzhou 450001, Henan, China; pengjj1996@163.com (J.P.); luanwangzzu@163.com (L.W.); zzuwangmengge@163.com (M.W.); durui1235@126.com (R.D.); 2 Henan Province Collaborative Innovation Center of New Drug Research and Safety Evaluation, Zhengzhou 450001, Henan, China; 3 State Key Laboratory of Esophageal Cancer Prevention & Treatment, Zhengzhou University, Zhengzhou 450052, Henan, China

**Keywords:** *lycium barbarum* polysaccharide, *Saccharomyces cerevisiae*, synthetic biology, Goji Berry

## Abstract

The fruit of *Lycium barbarum* L. (goji berry) is used as traditional Chinese medicine, and has the functions of immune regulation, anti-tumor, neuroprotection, anti-diabetes, and anti-fatigue. One of the main bioactive components is *L. barbarum* polysaccharide (LBP). Nowadays, LBP is widely used in the health market, and it is extracted from the fruit of *L. barbarum*. The planting of *L. barbarum* needs large amounts of fields, and it takes one year to harvest the goji berry. The efficiency of natural LBP production is low, and the LBP quality is not the same at different places. Goji berry-derived LBP cannot satisfy the growing market demands. Engineered *Saccharomyces cerevisiae* has been used for the biosynthesis of some plant natural products. Recovery of LBP biosynthetic pathway in *L. barbarum* and expression of them in engineered *S. cerevisiae* might lead to the yeast LBP production. However, information on LBP biosynthetic pathways and the related key enzymes of *L. barbarum* is still limited. In this review, we summarized current studies about LBP biosynthetic pathway and proposed the strategies to recover key enzymes for LBP biosynthesis. Moreover, the potential application of synthetic biology strategies to produce LBP using engineered *S. cerevisiae* was discussed.

## 1. Introduction

There are more than 80 *Lycium* species in the world, and they are widely distributed in Asia, Europe, North America, and other regions [1]. Among them, *Lycium barbarum* L. is a perennial deciduous shrub, and its fruits, goji berries, have been used as one of the traditional Chinese drugs [2,3]. Currently, goji berry is recorded in the Pharmacopoeia of the People’s Republic of China [4,5]. Goji berry has nourishing effects on the kidneys and lungs [6]. It is regarded as a nourishing Chinese medicine, and can be used as medicine and food [7]. Goji berry has diverse biological activities, including anti-inflammatory [8], anti-tumor [9], anti-oxidation [7], anti-aging [10], hypoglycemic [11], and hypolipidemic [12].

Goji berry is rich in nutrition and abundant in natural products, such as *L. barbarum* polysaccharide (LBP), betaine, flavone, and vitamin [3]. Among its bioactive components, LBP composes 5%–8% of the dried goji berry [13]. LBP is a water-soluble complex with carbohydrate chains and proteins; the carbohydrate chains are mainly composed of six saccharides, including arabinose, galactose, glucose, rhamnose, mannose, and xylose, which accounts for about 70% of all the saccharides [14,15]. LBP can modulate gut microbiota to improve nutrient utilization and health [16,17]. Nowadays, LBP is mainly extracted from goji berry. It takes one year to obtain goji berry, and climate, diseases as well as insect pests might affect the yield of goji berry [18]. Moreover, planting areas for high-quality goji berry with high-levels of LBP are limited. Besides, fresh goji berry is highly perishable, which limits the acquisition of high-quality LBP [19,20]. Extraction of LBP from goji berry is unable to meet the rapidly increasing commercial LBP demands. Therefore, it is of great interest to find other sustainable and stable LBP supplies [7].

With the rapid development of yeast synthetic biology, *Saccharomyces cerevisiae* and other yeasts have been used as cell factories for the production of plant natural products [21]. One advantage of producing polysaccharides using yeast cell factories is that the production is not affected by seasons, regions, and pests [22]. *S. cerevisiae* is generally recognized as a safe microorganism (GRAS) and has been widely used in food and drug production. Some plant natural products have been produced in engineered *S. cerevisiae*, such as cocoa butter and ginsenosides [23,24,25,26,27,28,29,30,31]. Therefore, *S. cerevisiae* is an ideal microbial host to produce LBP. Introducing the LBP biosynthetic pathway and rewiring the metabolic pathway of *S. cerevisiae* might provide a green and feasible way for LBP production [32,33].

## 2. LBP Biosynthetic Pathway in *L. barbarum*

LBP is constituted of six saccharides of α-(1→4)-GalA, α-(1→6)-Glc, β-(1→3)-Galp, β-(1→6)-Galp, α-(1→5) -Ara, and β-(1→4)–Galp. Enzymatic selectivity is essential to combine the diverse saccharides into LBP. After the glycosidic bond is formed, the stereochemistry of LBP can be retained or reversed by glycosyltransferases [34,35,36]. In fact, multiple metabolic pathways might participate in LBP biosynthesis in goji berry, including the galactose metabolism pathway, tricarboxylic acid cycle, glyoxylic acid cycle, propionic acid metabolism, amino sugars metabolism, starch metabolism, and sucrose metabolism. These pathways provide precursors for the anabolism of various carbohydrates [37,38]. Among these pathways, sucrose synthetic pathway is mainly responsible for plant cell wall formation and biomass accumulation. Sucrose phosphate synthase (SPS) and sucrose phosphate phosphatase (SPP) are the two essential enzymes in sucrose biosynthesis [39]. In addition, the acid invertase (AI), sucrose synthase (SS), and SPS involved in sucrose metabolism are the key enzymes of the plant carbon metabolic pathway [40]. Sucrose synthesis is closely associated with the metabolism of amino sugar, nucleotide sugar, and galactose [41]. The fruits of goji berry mainly synthesize glucose and fructose, and the LBP content is mainly determined by fructose content in goji fruits.

**Figure 1:**
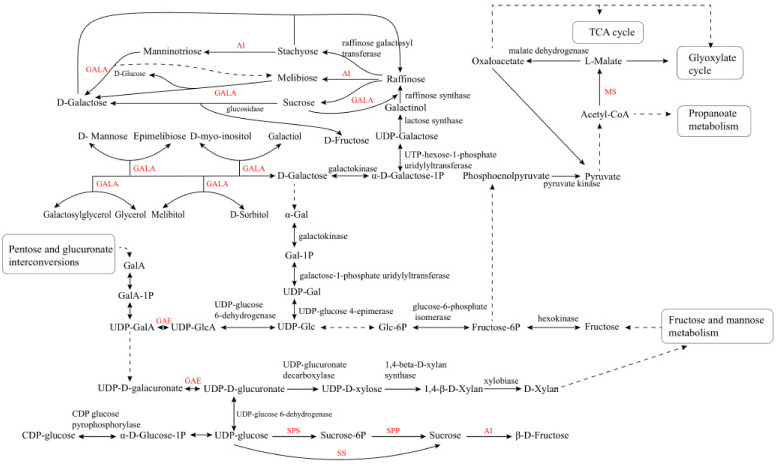
Metabolic pathways of saccharides in goji berry. AI, acid invertase; GAE, UDP-glucuronate 4-epimerase; GALA, α-galactosidase; MS, malate synthase; SPS, sucrose phosphate synthase; SPP, sucrose phosphate phosphatase; SS, sucrose synthase.

The α-galactosidase (GALA) participates in several steps of galactose metabolism. These carbohydrate anabolic pathways are the prerequisite for LBP synthesis, and they can regulate carbohydrate accumulation. UDP-glucose pyrophosphorylase (UGP) is an essential enzyme involved in carbohydrate metabolism, which can affect normal cell development, polysaccharide synthesis, and stress response in *S. cerevisiae* and other fungi [42,43]. The key enzymes in the LBP synthetic pathway, UDP-glucuronate 4-epimerase (GAE), malate synthase (MS), and α-galactosidase (GALA), have not been characterized yet [37]. These key genes play important roles in the synthesis of LBP, and the information might be obtained by analyzing the transcriptome of goji berry (Figure 1).

## 3. Mining of Key Enzymes for LBP Biosynthesis

The transcriptome of goji berry can help recover genes in the LBP biosynthetic pathway. Several studies about transcriptome of goji berry have been applied. Wang et al. prepared the leaf transcriptome of goji berry and elucidated the mechanisms of carotenoid biosynthesis in goji berry [44]. Chen et al. obtained 139,333 predicted genes of goji berry, and they identified genes in the flavonoid and taurine biosynthetic pathways [45]. Ma et al. analyzed the transcriptome of goji berry and identified the candidate genes involved in sugar metabolism under elevated CO_2_, which helped recover the LBP biosynthetic pathway [37]. However, current omics data in public databases have not recovered the whole LBP biosynthetic pathway in goji berry.

**Figure 2:**
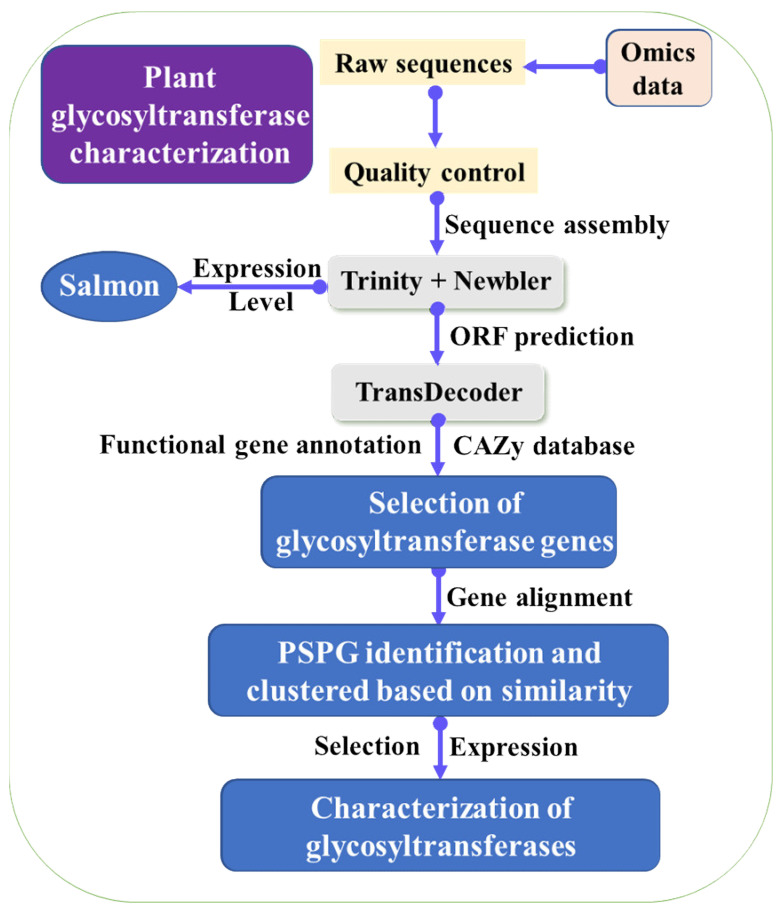
Strategy used for identification and characterization of glycosyltransferase genes from transcriptomic data.

To explore the key enzymes in the LBP biosynthetic pathway, collecting fresh tissues of goji berry at different growth phases and different planting areas are necessary. Sequencing of these fresh tissues would give an array of transcriptome data, and de novo assembly of these data would obtain full gene profiles of goji berry. Further transcriptome comparison analysis would give insights into the details of the LBP biosynthetic pathway (Figure 2). To predict the key enzymes in LBP synthetic pathways, phylogenetic, gene similarity network analysis, and other bioinformatic methods can be used to identify the candidate key genes [46]. Diverse glycosyltransferase genes were identified from public omics data by using phylogenetic analyses and plant secondary product glycosyltransferase (PSPG) homolog [28,29,30]. Thus, finding homologous in goji genomes is possible to predict potential glycosyltransferases for LBP synthesis. As the sugar metabolic pathway is complex, characterizing candidate LBP synthetic genes identified with bioinformatic strategies will help identify efficient enzymes for LBP biosynthesis in engineered yeasts.

The glycosyltransferases can determine saccharide compositions and arrangements of the LBP. The bioinformatic strategy was used for the recovery of glycosyltransferases functioned in ginsenoside biosynthesis. The omics data in the public database were downloaded and assembled. The open reading frames (ORFs) were predicted and annotated with the help of CAZy and other databases. The candidate glycosyltransferase genes were selected for sequence alignment. Only predicted glycosyltransferase genes with more than 1320 bp and PSPG motif were used for further analysis. The glycosyltransferase genes were clustered based on the similarity, in order to reduce the redundancy. Besides, gene expression level can be determined at different growth stages, which can help identify real glycosyltransferase genes. The identified potential glycosyltransferase genes for ginsenosides were selected for expression. The enzymatic characterization of the glycosyltransferases was identified (Figure 2), and the characterized glycosyltransferases were used for adding activated sugars to the substrates [28,29,30,31]. Recently, another two cellulose synthases were identified to function as glycosyltransferases in saponin biosynthesis [47,48], showing identification of glycosyltransferases for LBP synthesis is complex. Nowadays, the use of gene similarity and network analysis have been widely used for gene identification [46]. 

## 4. *S. cerevisiae* is an Efficient Cell Factory for the Production of Plant Natural Products

Polysaccharides are nontoxic and natural biodegradable biopolymers. Normally, the main components of plant cell wall and other biomass are polysaccharides. Besides, the extracellular polysaccharides play essential roles in biofilm formation. It is possible to use microorganisms to produce polysaccharides. *S. cerevisiae* is a popular organism for industrial-scale production of plant natural products, such as artemisinic acid (the precusor of artemisinin), ginsenosides, and cocoa butter [49,50,51]. Genetic engineering and synthetic biology strategies, including homologous recombination and CRISPR-Cas9 system, are easy to implement in *S. cerevisiae* [52,53]. 

As an ideal microbial cell factory for the production of plant natural products, *S. cerevisiae* is often used to characterize key genes of plant natural products. The cell wall of *S. cerevisiae* is mainly composed of glucan and mannose [54,55]. Furthermore, arabinose, rhamnose, xylose, and galactose can be synthesized in *S. cerevisiae* directly [56,57,58,59]. *S. cerevisiae* can use cheap substrates and expressing key LBP synthetic genes in *S. cerevisiae* are easy, therefore, converting cheap sugar substrates into LBP are possible.

Levan is a bacterial extracellular polysaccharide, and it can be used as prebiotic fibers in the production of functional foods and pharmaceutical formulation. Franken et al. engineered *S. cerevisiae* for the biosynthesis of levans. In order to synthesize levan, an invertase of *S. cerevisiae* was deleted to build an invertase null *S. cerevisiae* mutant. The levansucrase expressed in invertase null *S. cerevisiae* mutants or strains with sucrose accumulation strains led to the intracellular levan accumulation [60]. Co-expression of the levansucrase and the spinach sucrose transporter achieved hyper-production of extracellular levans [61]. Overexpression of the levansucrase gene of *Rahnella aquatilis* in *S. cerevisiae* and enhancing its secretion by expression with a secretion signal resulted in the final levan titer of 76 g/L in a 50-L fermenter [62]. The substrate sucrose conversion efficiency is 80% and the production rate is 3.17 g/L/h [62]. The β-Glucan is one of the main components of yeast. Overexpression of *PGM2* (encoding phosphoglucomutase) and *RHO1* (encoding a GTPase for activating glucan synthesis) enhanced β-Glucan production in *S. cerevisiae*. Optimization of the culture condition further led to a >53.1% increase [55]. Moreover, *S. cerevisiae* was discussed as one potential producer of chitosan, which is a β-1,4-linked glucosamine polymer [63]. 

## 5. Engineering Strategies for High-Level LBP Production in *S. cerevisiae*

**Figure 3:**
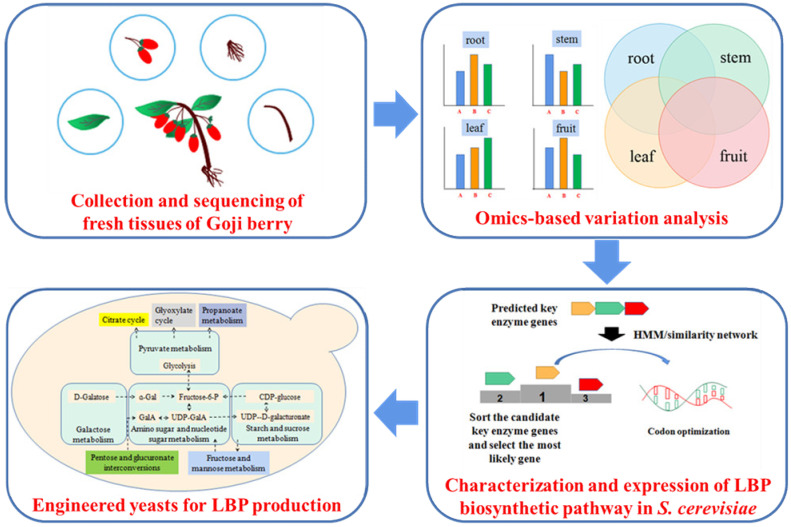
Construction of the *Lycium barbarum* polysaccharide (LBP) metabolic pathways in *Saccharomyces cerevisiae* through extraction of total RNA from different tissues of goji berry, analyzing the different expression strength in different parts. Using HMM and gene similarity network to analyze the predicted key enzymes, optimize the codons of the key enzyme would lead to the LBP production in engineered *S. cerevisiae* using synthetic biology strategies.

With the rapid development of the next-generation sequencing technology, plant transcriptome and genomic data were used to determine key genes in the LBP pathway, such as the integration of transcriptomic and metabonomic strategy for gene identification, and the variation analysis of different omics data [64]. AI and SPS are the key enzymes involved in sucrose metabolism. AI breaks down sucrose into glucose and fructose, and SPS catalyzes the synthesis of sucrose [48,65]. Balance expression of these genes in *S. cerevisiae* may help to synthesize LBP. Using bioinformatics and systems biology, we may characterize unknown key enzymes of GAE, MS, and GALA functioned in complex natural product biosynthesis (Figure 3) [66,67]. The identification of genes in the LBP biosynthetic pathway is the first step for LBP biosynthesis (Figure 3). Though LBP biosynthetic genes of goji berry might be the most efficient ones for LBP biosynthesis, the genes of goji berry might not be well expressed and functioned in *S. cerevisiae*. Codon-optimized and engineering of the key enzymes can possibly help obtain efficient enzymes for LBP biosynthesis in *S. cerevisiae*. 

When the engineered *S. cerevisiae* strain can synthesize LBP, selecting suitable substrates, enhancing the supply of cofactors and precursors, and the heterologous expression of key genes will direct the metabolic flux to LBP synthesis and improve the production of LBP. For industrial-scale production, optimization of yeast cultivation medium and conditions might further increase LBP production in *S. cerevisiae*. Some plant natural products produced with engineered yeasts, such as artemisinin, have been approved for medicinal use [43,44], but using products derived from engineered microorganisms as foods is rarely reported. Further demonstrating the safety of products derived from engineered microorganisms and discussing the possible revision of the regulatory rules are essential before providing engineered yeast-based products to consumers. As *S. cerevisiae* is GRAS, the *S. cerevisiae* biomass might be directly used as prebiotics for poultry or other animals without further bioprocess. In the future, the synthetic biology will help introduce the redesigned LBP biosynthetic pathway in yeast and lead to the high-level production of LBP in the future [68].

## 6. Conclusions and Future Perspective

As the main bioactive component, LBP has a variety of pharmacological effects, but its supply depends on the extraction from goji berry. Production of LBP by engineered *S. cerevisiae* provides a possible green strategy for LBP supply. The completed synthetic pathways and key enzymes are unclear now, which limits yeast production of LBP. Finding the key enzymes and designing the intact pathways using omics data are possible now. In the near future, construction of an optimized redesigned LBP synthetic pathway in *S. cerevisiae* will lead to large-scale production of LBP, which would provide a sustainable way for LBP supply.

## Data Availability

Not applicable.
